# F86. SOCIAL COGNITION IN HOMICIDE OFFENDERS WITH SCHIZOPHRENIA

**DOI:** 10.1093/schbul/sby017.617

**Published:** 2018-04-01

**Authors:** Katharina Engelstad, Bjørn Rishovd Rund, Anne-Kari Torgalsboen, Anja Vaskinn

**Affiliations:** 1 Vestre Viken Hospital Trust; 2 University of Oslo; 3 Oslo University Hospital

## Abstract

**Background:**

Schizophrenia is characterized by impairments in social and non-social cognition that act as strong predictors of the poor outcome of the disorder, including violence. In fact, it has been suggested that the inclusion of (social) cognition in risk assessment tools may increase their accuracy. A premise for this line of thinking is that individuals with schizophrenia and a history of violent offences should present with a different social cognitive profile than individuals with schizophrenia without such a history. In this study, we compare social and non-social cognition in homicide offenders with schizophrenia (HOS) and in individuals with schizophrenia without a history of interpersonal violence (non-HOS).

**Methods:**

Twenty-six HOS and 28 non-HOS were included. They underwent a comprehensive clinical and neuropsychological assessment protocol where the MATRICS Consensus Cognitive Battery (MCCB) and four tests of social cognition were applied. Facial emotion perception was assessed with Pictures of Facial Affect (PFA) where portrait photographs of people expressing one of six emotions are presented. Emotion perception from bodies was measured with Emotion in Biological Motion (EmoBio), a point-light task consisting of short movie clips where lighted dots, indicative of one of four emotions, move across the computer screen. Theory of mind (ToM) was indexed by two tests. The Hinting Task consists of ten short stories where a hint is dropped. The ecologically valid Movie for the Assessment of Social Cognition (MASC) shows four characters who meet for dinner. The movie is stopped several times, and the test taker is instructed to answer questions about the thoughts, intentions and emotions of a given character. This yields scores for cognitive and affective ToM. In addition, the multiple-choice response format provides information on overmentalizing and undermentalizing errors.

**Results:**

Preliminary analyses, using independent samples t-tests, showed significant differences for the overall scores on the EmoBio and MASC tests, with non-HOS outperforming HOS participants. Follow-up analyses for the EmoBio test revealed no statistically significant differences for any specific emotion, although the group difference for the recognition of happy, fearful or neutral body movements approached a medium effect size. On the MASC test, HOS presented with reduced cognitive and affective ToM, compared to non-HOS, and also committed more undermentalizing errors. These effect sizes were large.

**Discussion:**

Our preliminary analyses show that homicide offenders with schizophrenia have reduced emotion perception and theory of mind compared to individuals with schizophrenia without a history of interpersonal violence. This lends support to the idea that social cognitive deficits can be a risk factor for interpersonal violence in persons with a diagnosis of schizophrenia.

